# A cascade of care for people with epilepsy: learning from “HIV/AIDS 90-90-90”

**DOI:** 10.12688/gatesopenres.13043.2

**Published:** 2019-08-06

**Authors:** Farrah J Mateen

**Affiliations:** 1 Department of Neurology, Massachusetts General Hospital, Boston, MA, 02114, USA

**Keywords:** epilepsy, public health, child health, HIV/AIDS, health policy, health planning

## Abstract

Epilepsy is now more prevalent in many countries than HIV/AIDS. Building upon the advances of global policymaking for HIV/AIDS and creating a framework for countries and organizations to monitor progress in epilepsy care will help direct and justify much-needed novel programming. Given the clarity of the HIV/AIDS care continuum model and the UNAIDS 90-90-90 targets, I propose this same approach to the cascade of care could be used as a viable framework for people with epilepsy. In this model, the targets of success include (1) ensuring 90% of all people with epilepsy are aware of their diagnosis as a brain disorder, (2) starting 90% of people with epilepsy on quality controlled, appropriately chosen and well stocked antiepileptic drugs, and (3) achieving seizure freedom in 70% of those treated. At least 90% of all people with epilepsy must also be linked to and retained in appropriate care. Although the precise numbers may be debated, this cascade of care approach will assist in deconstructing the barriers to epilepsy care in populations better than the more familiar concept of the epilepsy treatment gap. These reflect concrete goals for health systems for epilepsy care that, if achieved, could lead to seizure freedom for the many people in lower income countries living with poorly controlled epilepsy.

## Background

Epilepsy is an important cause of chronic disability and a preventable cause of early mortality in low- and middle-income countries (LMICs). More than 1% of the population in LMICs, >60 million people, suffers from epilepsy ^1^. Phenobarbital, the oldest antiepileptic medication still in use today, was discovered in 1912. Phenobarbital costs 1 to 2 US cents per day or <5 USD per year and remains the drug of choice for several presentations of epilepsy. Four additional older antiepileptic drugs are commonly found on the World Health Organization’s Essential Medicines List and typically cost <50 cents per day.

 Epilepsy is an exemplary disease for health systems planning for brain disorders. Epilepsy presents across the lifespan, with the predominance of first presentations in childhood and in the elderly. The stigma of epilepsy, including its formal and informal prohibitions on school attendance, employment, and marriage in some societies, emphasizes it as an important challenge for the global public health community. Medically, it represents a final common manifestation of a myriad of possible causes: genetic conditions, developmental conditions, central nervous system infections, head trauma, stroke, and sometimes defies clear explanation of its etiology. This is typical of several neurological disorders in which etiologies may reflect the so-called “triple burden” of communicable, noncommunicable, and traumatic disorders. Access to diagnostic services for epilepsy, such as electroencephalogram and neuroimaging, enhances the diagnostic clarity of epilepsy, but the absence of infrastructure in LMICs does not preclude antiepileptic medication treatment. Women of childbearing potential represent a special treatment group since some antiepileptic medications should be avoided during pregnancy, especially valproic acid, given the risk of this medication causing congenital malformations including neural tube defects.

## Updating the approach to epilepsy treatment: a cascade of care

Prior framing of the global epilepsy challenge was through the epilepsy treatment gap ^2^, or the number of people with epilepsy (PWE) who are eligible for but not taking an antiepileptic medication. This gap reaches up to 90% in LMICs ^3, 4^. Meanwhile, a “zero” treatment gap remains unattainable, even in high-income settings. In this way, “getting to zero” is not a realistic goal for epilepsy care as it would be for infectious diseases, which could be eliminated or even eradicated. Although demonstration projects have shown important progress in the number of PWE who are able to become seizure free or reduce their seizure burden in lower income settings ^5^, even more can be done to detail the ways in which seizure freedom has occurred. Targeted epilepsy initiatives, such as the WHO Global Campaign on Epilepsy ^6^ *What you can do* can be evaluated with more precision.

 Using the treatment gap approach, essential steps in the care pathway of PWE have been overlooked. Since epilepsy is both a clinical problem and a matter of global policy, it requires metrics to optimize care and achieve population-based outcomes. Although countries may be meeting treatment gap goals, many PWE are not adequately diagnosed by seizure type. Some are treated with an inappropriate choice of antiepileptic medication. And in spite of adequate medication adherence, seizure freedom for many PWE may be difficult to attain due to inadequate dosing as well as limited quality and inconsistent supplies of antiepileptic medications ^4, 7– 9^.

 As HIV prevalence rates drop in many countries, epilepsy may be more prevalent in many countries than HIV/AIDS. People living with HIV/AIDS have benefitted from global advocacy, political will, and dedicated and sustained financial investments. Private-public partnerships and supranational agencies have brought light to the extreme tragedy of the HIV epidemic. This was achieved in spite of the stigma of HIV/AIDS and the disproportionate burden of HIV/AIDS in resource-limited settings and vulnerable populations. 

 The same efforts have not been made in epilepsy, an ancient disease, that can learn from the progress of HIV/AIDS. Building upon the progress of global policymaking for HIV/AIDS and creating a framework for countries and organizations to monitor progress in epilepsy care will help organize and justify novel programming. It may not achieve the stature of HIV/AIDS programming, but a framework for identifying the steps in epilepsy care pathways can be realized. Given the clarity of the HIV/AIDS care continuum model and the UNAIDS 90-90-90 targets, I propose this same approach to the cascade of care ^10^ could be used as a viable framework for PWE. In the HIV model, the measured targets include (1) ensuring 90% of all people with HIV infection know they are infected, (2) starting 90% of infected people on antiretroviral therapy, and (3) achieving viral suppression in 90% of those treated. At least 90% of all people with HIV are also linked to and retained in care. By comparison, similar stepwise targets could be applied to epilepsy care:

(1) *Diagnosis* of epilepsy allows patients to be successfully given their medical diagnosis, distinct from supernatural causes but also distinct from primary psychiatric behavioral events, cardiac dysrhythmias, symptomatic hypoglycemia, and related conditions.
(2) *Linkage to epilepsy care* allows the establishment and organization of services for PWE - and the minimum standards for epilepsy care - including medication management, as well as access to neuroimaging, EEG services, and/or supportive laboratory studies such as antiepileptic drug levels ^11^.
(3) *Antiepileptic medication treatment* enables the management of seizures through efficacious, appropriately chosen and prescribed, available, accessible, and affordable medicines.
(4) *Seizure control and freedom* requires the antiepileptic medication or, in some cases, multiple medications to effectively reduce the number of seizures, ideally to zero in at least 2/3 of PWE ^12^, and increase the number of seizure-free days. Although not explicitly required, minimization of side effects such as sedation, would be optimal.

**Figure 1.**
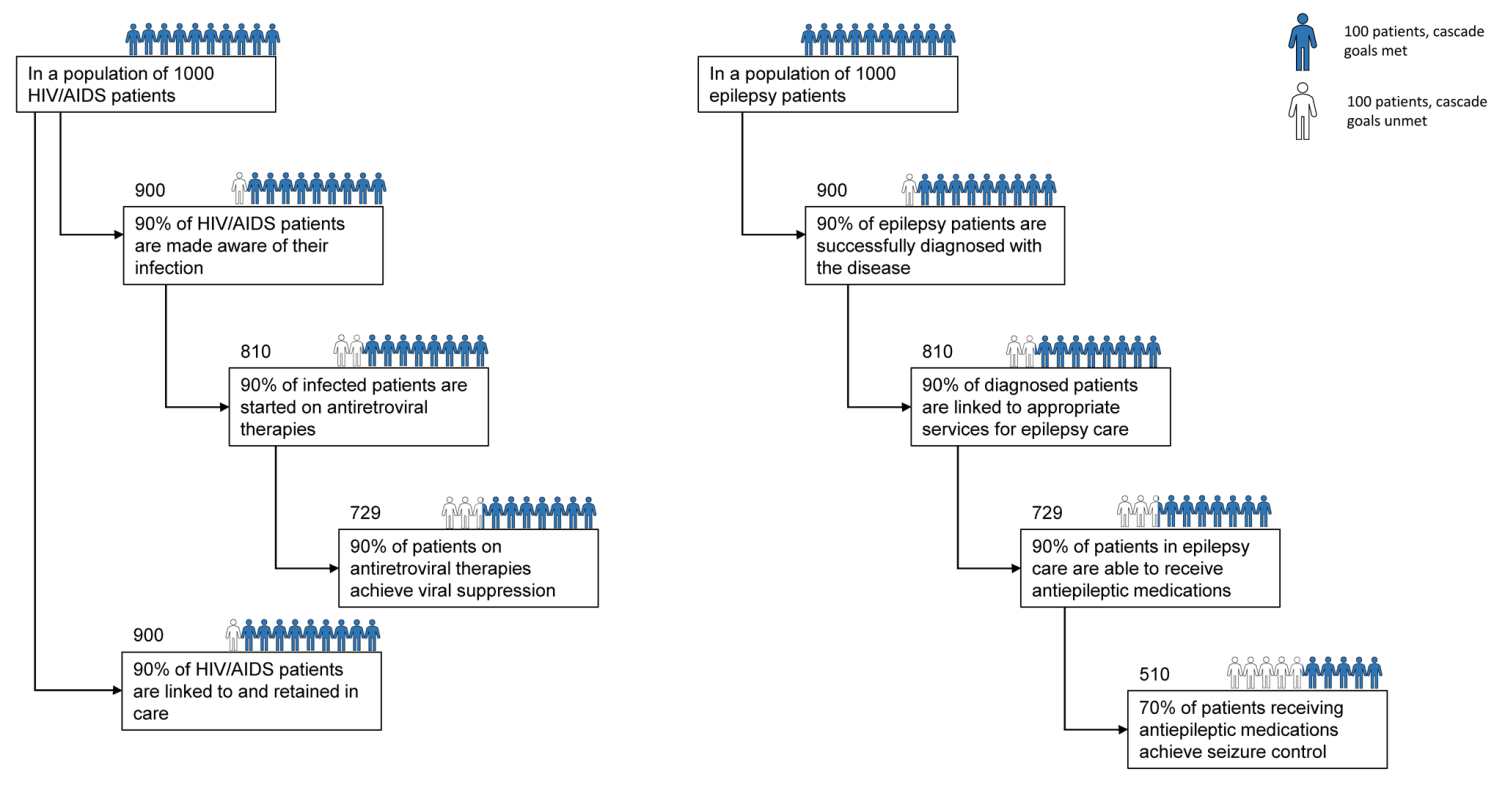
A comparison of the Cascade for Care of HIV/AIDS and Epilepsy in 1000 people with each condition.

This cascade of epilepsy care should have globally agreed targets, likely 90% of PWE being diagnosed; 90% of PWE linked and retained in care for epilepsy; and 90% of PWE who need an AED receiving it ( Figure 1). In addition, a reasonable goal of 70% of all PWE achieving seizure control should be targeted. This provides a fair comparison for services across higher and lower income settings and may indeed reflect, like in HIV/AIDS, that lower income countries are better able to implement cascades of care for more of their population. Although these precise numbers may be debated by the global community, they are goals that reflect actual processes of epilepsy care.

There are several barriers to measuring and realizing these metrics.

(1) *Diagnosis* of epilepsy can best be measured through community-based surveys in the population asking key survey questions. The lack of a distinct biomarker for epilepsy, such as a laboratory test, makes measurement often depend on semi-skilled providers.
(2) *Linkage to epilepsy care* is perhaps the most difficult step in the care pathway since it requires functionality of the health care system that will not be overwhelmed by new referrals or under-prepared to deal with a potential influx of patients if diagnoses are made. Long traditions of seeking non-physician healers in the care of PWE may be difficult to overcome ^13, 14^ and require dedicated efforts and educational initiatives to encourage patients to accept more scientific approaches. Counting of PWE who are not linked to care can be difficult.
(3) *Antiepileptic medication treatment* is realizable but there are insufficient efforts to make medications universally available, accessible, and affordable. Treatment of at least 70% of PWE will require non-governmental organizations, governments, supranational organizations, and patients. Barriers to realization of medication provision in 2019 remain common including out-of-date essential medicines lists, the variable quality of medication supplies in LMICs, lack of appropriate supply chains, excessive regulations on some medications, and high out-of-pocket costs to patients.
(3) *Seizure control and freedom* are both scientific and educational challenges. Barriers to achieving this metric include expertise on dosing medications, choosing medications appropriately, and having the time and resources to adequately educate patients. It requires addressing causes of medication-resistant epilepsy including preventable causes such as neurocysticercosis, vaccine-preventable perinatal infections, and many cases of preterm birth. It includes changing the behavioral pattern of taking a drug temporarily, as is common for an infectious disease, to taking a medication constantly and potentially lifelong. Additional barriers include the lack of epilepsy surgery opportunities for many LMICs and lack of access to an expanded list of newer scientifically proven antiepileptic medications. Although newer medications may be similarly efficacious to older medications, minimization of side effects and improving the range of options in locations without laboratory capacity can be particularly important.

## Conclusions

Epilepsy is a medically complex and historically poorly understood condition across cultures worldwide. In lower-income countries, neurologists are present in staggeringly low proportions. However, the metrics of achievement for epilepsy care can be made clearer and therefore can become achievable. Efforts to improve epilepsy care require more than appropriate metrics. Investment, both intellectually and financially; political interest and determination; establishment of a trained and capable health care workforce for epilepsy care; and consistently supplied antiepileptic drugs that are not prone to stock outs, poor quality, and market interests are all within our global capability. Downstream measurements of care at the level of the individual person with epilepsy can one day include the amount of out of pocket health expenditures, catastrophic health expenditures due to epilepsy, and missed opportunities such as schooling, work, and social functioning. Disaggregating these barriers to epilepsy treatment can inform the implementation of solutions and ultimately come full circle and “close” the more familiar “epilepsy treatment gap.”

## Data availability

No data are associated with this study.
