# Supplementary material for: A cascade of care for people with epilepsy: learning from “HIV/AIDS 90-90-90”
Source: Gates Open Res. 2019 Aug 6;3:1502. Originally published 2019 Jun 26. [Version 2] doi: 10.12688/gatesopenres.13043.2 (PMC6712888; doi:10.12688/gatesopenres.13043.2)
Supplement: Peer review history

# Peer review: Reviewer response for version 2

**DOI:** 10.21956/gatesopenres.14177.r27641

**Published:** 2019-08-23

**Authors:** W. Allen Hauser

**Affiliations:** 1 Gertrude H. Sergievsky Center, College of Physicians and Surgeons, Columbia University, New York, NY, USA

The author proposes a "cascade" of epilepsy care similar to that developed for the diagnosis and treatment of the HIVAIDS epidemic in LMIC countries. They now allude to the considerable efforts that have been undertaken over the past 20 years to address some of these problems and which seem to incorporate many of the basic strategies suggested. The goals set in the suggested cascade may be difficult to attain, but either as an independent effort or in concert with ongoing intervention strategies the proposal should be further explored. 

 One additional comment: under section 3 (4?), the points mentioned to be addressed (neurocysticercosis, vaccines, preterm birth) are causes of all epilepsy, not just medication-resistant epilepsy. Addressing these problems will have far reaching consequences - well beyond a goal of epilepsy prevention.

I confirm that I have read this submission and believe that I have an appropriate level of expertise to confirm that it is of an acceptable scientific standard.

# Peer review: Reviewer response for version 2

**DOI:** 10.21956/gatesopenres.14177.r27640

**Published:** 2019-08-08

**Authors:** Wolfgang Grisold

**Affiliations:** 1 Ludwig Boltzmann Institute for Experimental and Clinical Traumatology, Vienna, Austria

The points were addressed, and I can accept from my side.

I confirm that I have read this submission and believe that I have an appropriate level of expertise to confirm that it is of an acceptable scientific standard.

# Peer review: Reviewer response for version 1

**DOI:** 10.21956/gatesopenres.14161.r27519

**Published:** 2019-07-25

**Authors:** W. Allen Hauser

**Affiliations:** 1 Gertrude H. Sergievsky Center, College of Physicians and Surgeons, Columbia University, New York, NY, USA

The author proposes a novel approach to the problem of getting appropriate treatment worldwide for people with epilepsy. There are parallels of epilepsy and AIDS and suggests an approach similar to that used to address the AIDS's epidemic. This involved global advocacy, public-private partnerships, political will, and most importantly, sustained financial support for the programs which were implemented with specific metrics to allow ongoing evaluation of success. 

 It seems that many of these strategies have been implemented through the Global Campaign Against Epilepsy - a collaborative project between WHO and two international epilepsy organizations. It seems that some discussion of the successes and failures as well as mention of the long term activities related to these initiatives in the People's Republic of China would be worth including in this comment along with a review of the 2015 WHO recommendations.

 The treatment gap is at this point an ill-defined concept and I agree that it is a poor metric (without definition) to measure success of a program. I do not agree that it has been the sole metric used to assess interventions to improve care with epilepsy in the past. I don’t see the term mentioned in the guidelines. The development of a 90, 90, 90 metric to measure success is intriguing however and could be further developed although the actual measurement will be complicated.

 A couple of minor points: I have difficulties identifying other neurological conditions that have the scope of antecedents of epilepsy. Can some be provided by the author?

 It seems that the process starts with identification. This is not as easy as with AIDS. As pointed out a modicum of training is needed to identify cases; more if it is necessary to identify seizure type.  

 While the list of essential antiseizure medications may not include all of the latest drugs, there is little evidence that they provide a control advantage over the currently listed medications. They probably have fewer side effects but even if this is the case, (there are virtually no comparative studies) the cost differential may not be justified.

I confirm that I have read this submission and believe that I have an appropriate level of expertise to confirm that it is of an acceptable scientific standard, however I have significant reservations, as outlined above.

## References

[1] : From global campaign to global commitment: The World Health Assembly's Resolution on epilepsy. *Epilepsia*.2015;56(11) : 10.1111/epi.13192 1651-7 10.1111/epi.13192 26391429

# Response

**Published:** 2019-08-01

**Authors:** Farrah Mateen

The author proposes a novel approach to the problem of getting appropriate treatment worldwide for people with epilepsy. There are parallels of epilepsy and AIDS and suggests an approach similar to that used to address the AIDS's epidemic. This involved global advocacy, public-private partnerships, political will, and most importantly, sustained financial support for the programs which were implemented with specific metrics to allow ongoing evaluation of success. 

*I agree with the reviewer here. More than enthusiasm and recognition will be needed; dedicated and sustained funding are requisite. I have a sentence to this effect in the revised manuscript. This piece is meant to provide metrics for measuring whether efforts can be monitored, evaluated and even compared.*

It seems that many of these strategies have been implemented through the Global Campaign Against Epilepsy - a collaborative project between WHO and two international epilepsy organizations. It seems that some discussion of the successes and failures as well as mention of the long term activities related to these initiatives in the People's Republic of China would be worth including in this comment along with a review of the 2015 WHO recommendations.

*I am trying not to focus on any one particular country in this position piece although I recognize some regions in some countries have made major progress. I now cite the WHO Information Kit on Epilepsy as a pragmatic resource and example of work and progress to date.*

The treatment gap is at this point an ill-defined concept and I agree that it is a poor metric (without definition) to measure success of a program. I do not agree that it has been the sole metric used to assess interventions to improve care with epilepsy in the past. I don’t see the term mentioned in the guidelines. 

*Although the WHO and its publications have been thought leaders for epilepsy, the recommendations are arguably insufficient. More can be done. Compared to other neurological diseases, such as poliomyelitis which have global campaigns backed by weekly updates, websites, multilateral partnerships, and governmental and supranational prioritization, I believe epilepsy has fallen behind and so this may be a new score card approach for some settings.*

The development of a 90, 90, 90 metric to measure success is intriguing however and could be further developed although the actual measurement will be complicated.

*Yes, I agree. It is not easy but it is an effort to begin this work.*

A couple of minor points: I have difficulties identifying other neurological conditions that have the scope of antecedents of epilepsy. Can some be provided by the author?

*I would suggest dementia and cognitive decline as one. Another could be neurodevelopmental delay. Another – to less extent – could be cerebrovascular disease. One may argue these have communicable, noncommunicable, and traumatic risk factors. I understand the reviewer’s point that epilepsy is particularly broad in its antecedents though.*

It seems that the process starts with identification. This is not as easy as with AIDS. As pointed out a modicum of training is needed to identify cases; more if it is necessary to identify seizure type.  

*Yes, there is no one definitive biomarker like in AIDS. The movement from a single virus to a neurological disorder is a challenge. Yet, the public health understanding of a high-prevalence, global, life-threatening condition, subject to stigma and in need of global attention made me wish to compare these two. This line of thinking is more of a public health and stakeholder perspective than from the medical diagnostic one. Although non-parallels can be found, I still hope the broader comparison is worthwhile.*

While the list of essential antiseizure medications may not include all of the latest drugs, there is little evidence that they provide a control advantage over the currently listed medications. They probably have fewer side effects but even if this is the case, (there are virtually no comparative studies) the cost differential may not be justified.

*That is true. However, cost is a matter of negotiation for some of these drugs rather than the scientific challenge; so that could be the next round of measured metrics – i.e. out of pocket payments and catastrophic health expenditures by people with epilepsy. I suspect I am similar to the reviewer here in that I am of the opinion that this is a solvable problem. With enough funding, political will, private-public partnerships, and increased and unrelenting advocacy of our community, surmountable barriers for epilepsy care could be overcome and the evidence base could be expanded to better address these issues.*

# Peer review: Reviewer response for version 1

**DOI:** 10.21956/gatesopenres.14161.r27463

**Published:** 2019-07-08

**Authors:** Wolfgang Grisold

**Affiliations:** 1 Ludwig Boltzmann Institute for Experimental and Clinical Traumatology, Vienna, Austria

This is interesting and stimulating, to compare the success of the HIV/AIDS campaign, with possible strategies to treat patients with epilepsy worldwide.

 Despite the success(es) at many levels, HIV remains a threat in many low income countries, and has not been resolved sufficiently. It may be misleading  to celebrate this as a success, as in many countries, in particular in Africa have completely unmet needs.

 The treatment of epilepsy also needs to break through many stigmata, misbeliefs and take the fact into consideration, that in Africa, only a small percentage of patients are seen by qualified health care professionals, and most (some estimate 80%) by healers.

 Finally, and Dr Mateen points this out, there are 2 more important factors: 1) the work force of HCP, 2) and the drug costs, which seem minimal to us, but may be a large amount for persons in need. Culturally, and this may also be a point to consider, the acceptance of "western" medicine, is often considered with mistrust and afterthoughts of colonialization.

 My summary is, that this paper is an important and interesting comparison, between the success of the HIV campaigns, and its possible use in epilepsy.

 I recommend to relativize the HIV success and consider the low income countries, tackle the cultural aspects (which may be prohibitive in accepting treatment), and finally give more consideration on HP workforce, costs and coverage.

I confirm that I have read this submission and believe that I have an appropriate level of expertise to confirm that it is of an acceptable scientific standard, however I have significant reservations, as outlined above.

# Response

**Published:** 2019-08-01

**Authors:** Farrah Mateen

This is interesting and stimulating, to compare the success of the HIV/AIDS campaign, with possible strategies to treat patients with epilepsy worldwide.

*I thank the reviewer for his comments.*

Despite the success(es) at many levels, HIV remains a threat in many low income countries, and has not been resolved sufficiently. It may be misleading to celebrate this as a success, as in many countries, in particular in Africa have completely unmet needs.

*I agree with this statement.  In order to not over-state the HIV/AIDS field’s achievements, I have used words such as “progress” instead of “success.” Although scientific and policy efforts have not prevented or cured HIV/AIDS and there remain many million people infected globally, the counterfactual situation to this pandemic being unaddressed is even worse. Although the work is by no means complete, especially for locations like Sub-Saharan Africa, there is much to be learned for people with epilepsy, and arguably, there have still been major successes in this work for HIV/AIDS.*

The treatment of epilepsy also needs to break through many stigmata, misbeliefs and take the fact into consideration, that in Africa, only a small percentage of patients are seen by qualified health care professionals, and most (some estimate 80%) by healers.

*This is almost certainly correct and in fact I have thought about this issue a great deal in recent work published on traditional medicine and epilepsy in Guinea and in Bhutan. This is a cross-cultural and international issue. As such, I have added a new statement on the barriers to epilepsy diagnosis in this paper and thank the reviewer for emphasizing this major challenge. I also include in some additional citations to underscore this point.* 

Finally, and Dr Mateen points this out, there are 2 more important factors: 1) the work force of HCP, 2) and the drug costs, which seem minimal to us, but may be a large amount for persons in need. Culturally, and this may also be a point to consider, the acceptance of "western" medicine, is often considered with mistrust and afterthoughts of colonialization.

*This is certainly the case in multiple geographic locations. The workforce and drug supply are additional metrics that need to be measured and could be part of an expanded framework, beyond the 90-90-90 (or 90-90-70 approach). The data on colonization and mistrust is a bit less clear and less available in the literature. Although there are definitely reports on mistrust, the literature is limited and this becomes harder for me to cite. Also, I wanted to avoid focusing on any one geographic region in this paper and take “lower income countries” as a collective.*  

My summary is, that this paper is an important and interesting comparison, between the success of the HIV campaigns, and its possible use in epilepsy. I recommend to relativize the HIV success and consider the low income countries, tackle the cultural aspects (which may be prohibitive in accepting treatment), and finally give more consideration on HP workforce, costs and coverage.

*Thank you for your ideas. I emphasize HCP and medications more in the revision. I hope this piece opens up a larger conversation on global epilepsy, an under-discussed topic compared to its substantial (and likely growing) burden.*
